# Mosquito communities and disease risk influenced by land use change and seasonality in the Australian tropics

**DOI:** 10.1186/s13071-016-1675-2

**Published:** 2016-07-07

**Authors:** Dagmar B. Meyer Steiger, Scott A. Ritchie, Susan G. W. Laurance

**Affiliations:** Centre for Tropical Environmental and Sustainability Studies (TESS) and College of Marine and Environmental Sciences, James Cook University, 4870 Cairns, Queensland Australia; School of Public Health, Tropical Medicine and Rehabilitative Sciences, James Cook University, 4870 Cairns, Queensland Australia

**Keywords:** Edge effects, Deforestation, Land use change, Mosquito community, Rainforest disturbance

## Abstract

**Background:**

Anthropogenic land use changes have contributed considerably to the rise of emerging and re-emerging mosquito-borne diseases. These diseases appear to be increasing as a result of the novel juxtapositions of habitats and species that can result in new interchanges of vectors, diseases and hosts. We studied whether the mosquito community structure varied between habitats and seasons and whether known disease vectors displayed habitat preferences in tropical Australia.

**Methods:**

Using CDC model 512 traps, adult mosquitoes were sampled across an anthropogenic disturbance gradient of grassland, rainforest edge and rainforest interior habitats, in both the wet and dry seasons. Nonmetric multidimensional scaling (NMS) ordinations were applied to examine major gradients in the composition of mosquito and vector communities.

**Results:**

We captured ~13,000 mosquitoes from 288 trap nights across four study sites. A community analysis identified 29 species from 7 genera. Even though mosquito abundance and richness were similar between the three habitats, the community composition varied significantly in response to habitat type. The mosquito community in rainforest interiors was distinctly different to the community in grasslands, whereas forest edges acted as an ecotone with shared communities from both forest interiors and grasslands. We found two community patterns that will influence disease risk at out study sites, first, that disease vectoring mosquito species occurred all year round. Secondly, that anthropogenic grasslands adjacent to rainforests may increase the probability of novel disease transmission through changes to the vector community on rainforest edges, as most disease transmitting species predominantly occurred in grasslands.

**Conclusion:**

Our results indicate that the strong influence of anthropogenic land use change on mosquito communities could have potential implications for pathogen transmission to humans and wildlife.

**Electronic supplementary material:**

The online version of this article (doi:10.1186/s13071-016-1675-2) contains supplementary material, which is available to authorized users.

## Background

The emergence and re-emergence of mosquito-borne diseases can often be linked to human land use changes such as deforestation, agriculture and urbanization [1–5]. These land use changes may influence disease prevalence and distribution by increasing breeding habitats, food resources, and changing vector-host relationships [4, 6–8]. Tropical deforested habitats are open, well lit and warmer compared to secondary and primary forests [9]. These characteristics may increase the survival and growth rates of mosquito larvae [4, 8, 10, 11]. Newly available habitats for mosquitoes, such as irrigation systems, dams and other water-holding bodies, have also enabled mosquitoes to spread into previously uninhabitable areas [12, 13].

A principle risk factor in the emergence of zoonotic diseases (diseases that transfer from other animals to humans) is the alteration of the vector-host relationship due to land use modification [14, 15]. This change in relationship occurs when a vector is introduced to a new habitat or exposed to a new host. Human infection with yellow fever virus in South America is one such example [16, 17]. Within their natural environment, the yellow fever virus (*Flavivirus* spp.) is mainly transmitted by *Hemagogus, Sabethes* and *Aedes* mosquitoes to monkeys in the rainforest canopy. After logging and land clearing, mosquitoes followed the canopy edge to the ground where they fed and infected humans [16–18].

Seasonality in the tropics can influence mosquito populations, as the duration of wet and dry seasons affects larval development and adult abundance. Wet season rains create more breeding habitats, and elevated humidity levels extend the lifespan of adults, thus prolonging disease transmission rates [3]. For example, dengue outbreaks regularly coincide with wet seasons in Brazil, Thailand and Australia [19–21].

Mosquito-borne diseases such as malaria, yellow fever and chikungunya are thriving worldwide, especially in the tropics. The tropical regions of Australia could also be vulnerable to these diseases as potential vectors are present and disease transmission could arise due to infected people entering the country [22–24]. For example, potential vectors that occur in tropical Australia are: *Anopheles farauti* and *An. annulipes* for spreading malaria [25], and *Aedes aegypti* for the transmission of yellow fever and chikungunya [26, 27]. Unfortunately, human populations in Australia’s tropical regions are not immune to the effects of mosquito-borne infections as attested by outbreaks of dengue, Ross River fever, Barmah Forest virus, Japanese encephalitis and Murray Valley encephalitis virus [28]. There is very little known about the ecology of these diseases or their vectors in the Australian tropics and if environmental change has influenced their prevalence.

Our study investigated the mosquito community structure and composition across an anthropogenic disturbance gradient of grassland, forest edge and forest interior habitats in the tropical lowlands of north Queensland, Australia. Our main objectives were to evaluate how mosquito abundance, number of species and species composition differed between the three habitat types and across seasons. Our study presents a template to assess how landscape disturbances are able to influence mosquito species composition and distribution in the tropics and how those changes may influence mosquito borne diseases.

## Methods

### Study area

**Fig. 1:**
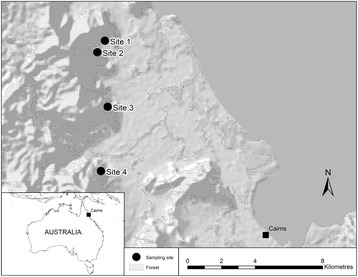
Map of study area and sampling sites. The study area, north of Cairns, Australia showing the four sampling sites which feature similar ecological habitats and environmental gradients

This study was conducted in the Wet Tropics bioregion of north eastern Australia. Study sites were located approximately 10 to 15 km north of Cairns city, (16°50’S, 145°41’E (Fig. 1), which provides an ideal setting for the analysis of mosquito response to land use changes as Cairns’ human population is growing and urban areas are expanding into agricultural and forest habitats. The population has more than doubled within 25 years (1981–2006) from 70,762 to 147,538. Furthermore, it is expected to be 1.4 to 1.7 times larger by 2031 [29], resulting in further land use changes [30]. Cairns is also an important tourist destination with an international airport and seaport for cruise liners and container ships; all of which have the potential to introduce exotic infectious agents into the country [31–34].

Annual rainfall is ca. 2000 mm yr^1^ and strongly seasonal with a wet season from December to May and a dry season from June to November. Temperature reaches an annual mean maximum and minimum of 29 °C yr^1^ and 20.8 °C yr^1^ respectively. The lowland evergreen rainforests within the study area are classified locally as Notophyll Vine Forests (Type 7) [35]. These forests contain trees that are 15–24 m in height with emergents such as *Acacia polystachya*, *Eucalytpus pellita* and *Eucalyptus tessellaris* on the ridges [35]. The forests have had some anthropogenic disturbances such as the removal of timber species [36], and fires that have escaped from cane farms [37]. The grasslands surrounding the forests were man-made and dominated by 2–4 m high non-native grasses, shrub species, and some pioneer rainforest trees.

### Sampling methods for mosquitoes

Field work was conducted between October 2011 and August 2012 at four sites with similar ecological habitats and environmental gradients. Every site was sampled four times; twice in the wet season and twice in the dry season. We captured adult mosquitoes from three different habitats: forest interior, forest edge and adjacent grassland. The distance between each of these habitats was at least 100 meters. Mosquitoes were collected using Center for Disease Control and Prevention (CDC) light traps (model 512, John W. Hock Company, Gainesville, Florida). The traps were modified by removing the light bulbs to avoid sample bias – as some mosquito species may be more attracted to light than others; to reduce attracting non-target insects that can damage mosquitoes; and to increase battery life [38]. All traps were run with 6 volt batteries. Six traps were set up in each habitat at each site resulting in 18 trap catches per site per sampling period. Traps were established along a transect at least 20 meters apart and were placed at a height of approximately 1.5 meters above ground level. The traps were baited with CO_2_ (2 kg dry ice per trap) in insulated containers, which were placed directly above the traps. Vaseline petroleum jelly was applied to suspension ropes to deter green ants (*Oecophylla smaragdina*) from reaching the caught mosquitoes. Traps were in operation for 24 h to ensure that both diurnal and nocturnal mosquito species were captured. After collecting the traps, species were stored in the insulated containers on the remaining dry ice and taken back to the laboratory where they were placed into freezers (-21 °C) for further storage.

### Mosquito identification

Mosquitoes were identified to species level using taxonomic keys [25, 39, 40] and with the assistance of taxonomic experts. The identification of mosquitoes is time consuming as it generally requires the keying out of each individual. Due to the large numbers of mosquitoes captured we applied a subsampling procedure that required the random selection of individuals for identification, this method was previously tested [41] and was found to accurately predict species diversity and maintain species proportions within the community.

### Statistical analysis

We assessed whether the subsampling technique accurately described species composition by comparing the relative abundance of one species (*Coquillettidia* nr*.crassipes*) in the subset with its actual abundance in the whole sample, by using Spearman’s rank order correlation. Only traps that had captured ≥ 80 mosquitoes were used which resulted in 30 traps being analysed. We only included traps with ≥ 80 mosquito captures due to the results of our pilot study which showed that cumulative species richness begins to plateau at 80 individuals.

We estimated the differences in mean abundance for each habitat type (forest interior, forest edge and grassland) and each season (wet and dry season) using a two-way ANOVA (independent factorial design with fixed factors). Variables were log-transformed to satisfy the assumptions of the residuals conforming to a normal distribution and in homogeneity of variances. A two-way ANOVA was also used to assess whether there was a difference in mean number of species between habitats and seasons. Data were not transformed prior to the analysis as statistical assumptions were met. The data for the early and late dry seasons, and the early and late wet season were pooled to derive the dry season and wet season data respectively. Rank-abundance diagrams distinguished changes in species dominance between habitats and seasons.

To evaluate if mosquitoes were sampled adequately under our sampling design, we constructed a species accumulation curve to display the cumulative number of species collected against the measure of sampling effort. The sampling effort is all data across the three habitats. Chi-square tests were applied to investigate whether the different mosquito tribes and subfamilies had habitat preferences.

To examine major gradients in the composition of mosquito communities and vector communities (vector communities consist of species which are able to transmit alpha-, flaviviruses and protozoans) between habitat types and seasons, we performed nonmetric multidimensional scaling (NMS) ordinations. Data were log (n + 1) transformed prior to analysis. Monte Carlo randomization tests (250 runs) were used to determine whether the ordination axes explained significantly more variation than expected by chance. A Bonferroni correction was used to reduce the likelihood of type II errors, where *P* = 0.15/x (x represents the number of mosquito species multiplied by two or three axes and 0.15 is the experiment wise error rate) [42]. Permutation-based nonparametric MANOVAs (PerMANOVAs) [43], followed by pairwise comparisons for significant results, were employed to distinguish differences in mosquito communities for the three habitat types.

Finally, we assessed whether there were differences between commonly captured mosquitoes (> 40 individuals) with similar ecological and biological characteristics by applying two-way ANOVA tests. Species were grouped according to the following three ecological and biological characteristics: geographical range in Australia, breeding environments and time of blood-feeding with the use of published life history and distributional data [25, 40]. The geographical range of species occurrence was divided into four groups: *very restricted* (species restricted to north Queensland only); *restricted* (species restricted to either north Queensland and Northern Territories or to Queensland and New South Wales); *medium* (northern Australia: Queensland, Northern Territories and Western Australia) and *wide* (all of Australia, except Tasmania). Mosquito species were further classified as using the following known breeding environments: *ground water* and *containers* (natural and artificial). Preferred time of blood-feeding of species was divided into *diurnal*, *nocturnal* and *crepuscular* (dawn and dusk). Geographical range data were square-root transformed to fulfill the assumptions of two-way ANOVAs.

We used SPSS statistical package (SPSS Statistics for Windows 22.0, Armonk, New York, USA) for most analyses, except for the ordination analyses and the PerMANOVAs for which we used PC-ORD (PC-ORD 6.0, MjM Software, Gleneden Beach, Oregon, USA). Where required, data was tested for normality by using the Kolmogorov-Smirnov tests and Levene’s tests for homogeneity of variances.

## Results

At the outset, we verified the accuracy of our subsampling method for estimating the relative abundance (proportion) of mosquito species. We found a strong positive correlation between the estimated and actual abundance of *Cq.*nr. *crassipes* in our samples (Spearman Rank Correlation *r*_s_ = 0.833, *P* < 0.001), which infers that our subsampling technique adequately estimated species composition.

### Mosquito captures

**Fig. 2:**
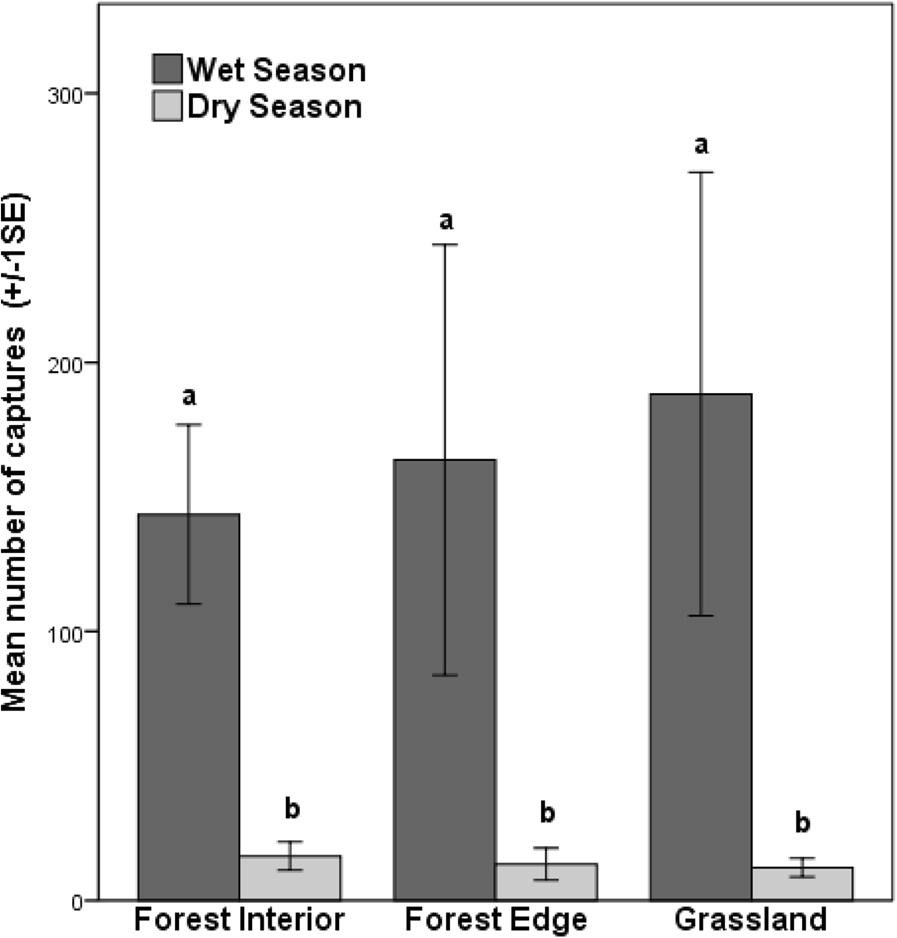
Abundance of mosquitoes in the three sampled habitats. The mean number of mosquitoes caught in forest interior, forest edge and grassland habitats during the wet and dry season. Mean values are similar in the three habitats but are significantly lower in the dry season than those in the wet season. Error bars denote the standard error. Different letters denote significant differences

In total, 12,854 mosquitoes were captured in 288 trap-nights across 16 grassland-edge-rainforest gradients. On average 93 ± 86 (mean ± SD) mosquitoes were captured per trap (Additional file 1: Table S1). Mosquito captures were quite evenly distributed across the three habitats as there was no significant difference between the mean captures per habitat (2-way ANOVA *F*_(2,18)_ = 0.109, *P* = 0.897). However, seasonality influenced mosquito abundance strongly. Thirteen times more mosquitoes were captured during the wet season than during the dry season sampling period (2-way ANOVA *F*_(1,18)_ = 65.555, *P* < 0.0001) (Fig. 2). No effect of interaction occurred between habitat and season (2-way ANOVA *F*_(2,18)_ = 0.150, *P* = 0.861).

We identified four tribes and two subfamilies of mosquitoes (Table 1) in the community. Members of the mosquito tribes Aedini and Mansoniini were found mostly in forest interior habitats (*χ*^*2*^ *=* 186.39, *df* = 1, *P* < 0.001 and *χ*^*2*^ *=* 30.26, *df* = 1, *P* < 0.001), whereas members of the tribe Culicini were predominantly captured in grassland habitats (*χ*^*2*^ *=* 92.57, *df* = 1*, P* < 0.001). No habitat preference was found for the Sabethini tribe (*χ*^*2*^ *=* 2.46, *df* = 1, *P* > 0.05). Most (95 %) individuals were in the subfamily Culicinae, with only 5 % in Anophelinae. Mosquitoes in the subfamily Culicinae were mostly captured inside forests (*χ*^*2*^ *=* 21.84*, df* = 1, *P* < 0.001) compared to mosquitoes in the subfamily Anophelinae which appeared to prefer open habitats, as they were significantly more abundant in grasslands compared to forest edges and forest interiors (*χ*^*2*^ *=* 75.20, *df = *1, *P* < 0.001).

**Table 1:** Mosquito abundance by tribe and subfamily from the three habitats in tropical Australia

		Forest interior		Forest edge		Grassland		Total		Chi-Square test (*df* = 2)	
Tribe/SUBFAMILY		n	%	n	%	n	%	N	%	*X* ^2^	*P*
Aedini		953	60.66	689	47.03	444	31.11	2086	46.74	186.39	< 0.001
Culicini		479	30.49	633	43.2	824	57.75	1936	43.38	92.57	< 0.001
Mansoniini		106	6.75	63	4.3	42	2.94	211	4.73	30.26	< 0.001
Sabethini		17	1.08	9	0.62	0	0	26	0.58	2.46	ns*
	Anophelinae	16	1.02	71	4.85	117	8.2	204	4.57	75.2	< 0.001
	Culicinae	1555	98.98	1394	95.15	1310	91.8	4259	95.43	21.84	< 0.001

*ns, not significant (*df* = 1)

### Species richness

**Fig. 3:**
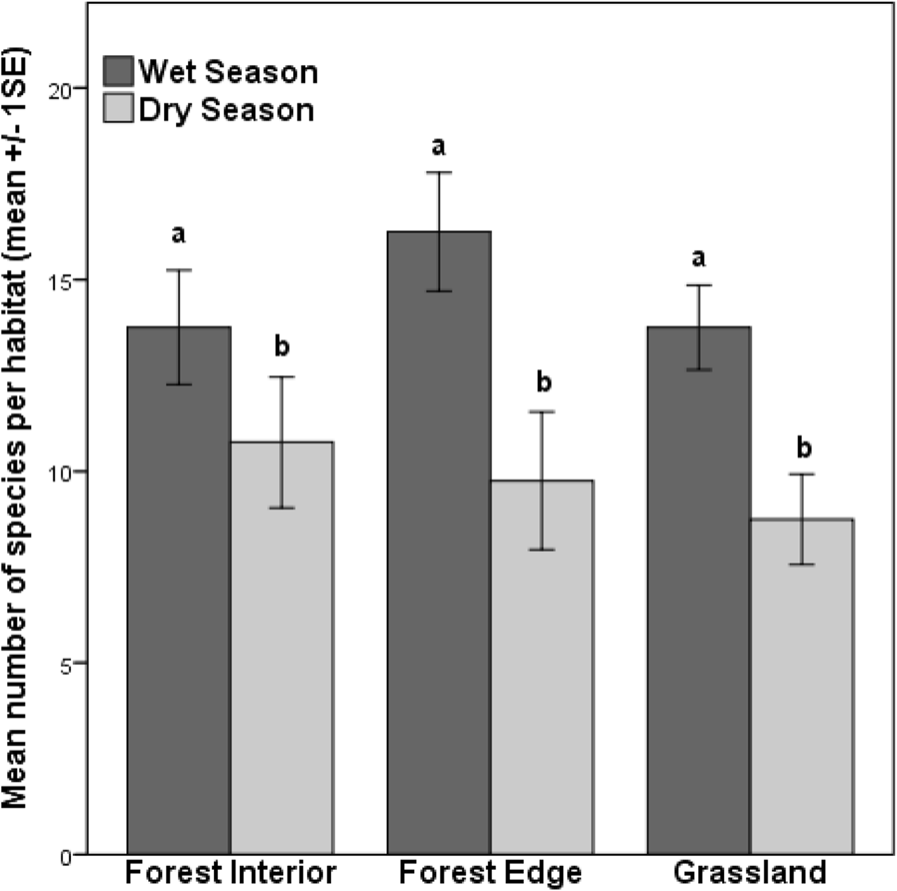
Mean number of mosquito species in the three sampled habitats. Mean number of mosquito species captured in forest interior, forest edge and grassland habitats during the wet and dry season in each habitat. Mean species richness was quite similar between the three habitats but significantly fewer species were captured in the dry season compared to the wet season, especially along forest edges and grassland sites. Error bars denote the standard error. Different letters denote significant differences

**Fig. 4:**
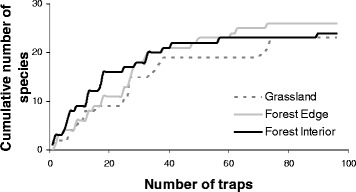
Species accumulation curves for sampled mosquitoes. Species accumulation curves for mosquitoes sampled from forest interior, forest edge and grassland habitats suggest that most common species were captured. The curves display adequate sampling effort for all habitats and indicate that further sampling would not have produced the discovery of more species, except for very rare ones

The mosquito community was very diverse with 29 species (27 species in the wet season and 24 species in the dry season) identified from 7 genera (Table 2; Additional file 1: Table S2, S3, S4, S5). The genera *Aedes* recorded the highest number of species (10 spp.), whereas *Culex* spp. were captured most frequently (1936 individuals). Four species dominated the total samples, together contributing to 74 % of the captures; *Culex annulirostris* (36 %), *Verrallina lineata* (16 %), *Aedes notoscriptus* (11 %) and *Aedes vigilax* (11 %). The most dominant species in the wet season were *Cx. annulirostris* and *Ve. lineata*. During the dry season*, Cx. annulirostris* was again the most abundant species, followed by *Ae. notoscriptus*. Some species, such as *Ae. alternans*, were only captured during the wet season and other species, such as *Ae. lineatopennis* were predominantly trapped during the dry season (Table 2). Mean mosquito species richness was similar in all habitats (2-way ANOVA *F*_(2,18)_ = 0.692, *P* = 0.514) but differed significantly between the seasons (Fig. 3). More species were captured in the wet season than in the dry season (2-way ANOVA *F*_(1,18)_ = 15.720, *P* = 0.001). There was no significant interaction between habitat and season (2-way ANOVA *F*_(2,18)_ = 0.692, *P* = 0.514). Species accumulation curves from our sampling design show the difference in species capture rate between the three habitats. The asymptotes of the curves suggest that our traps captured all the attracted species present in the habitats. However, it is possible that we did not trap either rare species or those not attracted to the traps or bait used (Fig. 4).

**Table 2:** Total number of each mosquito species collected from each habitat (FI = forest interior, FE = forest edge, GR = grassland) in north Queensland and the pathogens they may transmit

	Wet Season				Dry Season				
Species	FI	FE	GR	Total	FI	FE	GR	Total	Grand-total
*Aedes alboscutellatus* ^[?]^	10	7	14	31	24	12	0	36	67
*Aedes alternans* ^[a]^	1	5	4	10	0	0	0	0	10
*Aedes kochi* ^[a,d,e]^	2	30	26	58	3	3	8	14	72
*Aedes lineatopennis* ^[a,g]^	1	4	55	60	0	1	0	1	61
*Aedes notoscriptus* ^[a,b,d]^	144	119	32	295	97	62	34	193	488
*Aedes palmarum* ^[?]^	15	2	0	17	24	7	1	32	49
*Aedes quasirubithorax* ^[?]^	0	0	3	3	2	0	0	2	5
*Aedes quinquelineatus* ^[?]^	16	14	0	30	66	17	3	86	116
*Aedes tremulus* ^[a,b,]^	10	2	3	15	3	1	0	4	19
*Aedes vigilax* ^[a,b,d,e]^	86	106	108	300	45	57	71	173	473
*Anopheles annulipes* (s.l.) ^[a,c,d,e]^	1	0	4	5	3	3	5	11	16
*Anopheles bancroftii* ^[c,e]^	0	0	0	0	1	0	0	1	1
*Anopheles farauti* ^[b,c,e]^	8	58	99	165	3	10	9	22	187
*Coquillettidia* nr.*crassipes* ^[c,f]^	95	49	7	151	6	7	4	17	168
*Culex annulirostris* ^[a,b,d,e,g]^	390	456	544	1390	38	77	125	240	1630
*Culex bitaeniorhynchus* ^[b,e]^	0	1	0	1	0	0	0	0	1
*Culex cubiculi* ^[?]^	0	0	0	0	0	1	0	1	1
*Culex gelidus* ^[a,b]^	2	36	115	153	0	1	6	7	160
*Culex hilli* ^[?]^	12	18	2	32	6	5	0	11	43
*Culex pullus* ^[b]^	16	26	12	54	0	1	1	2	56
*Culex sitiens* ^[e]^	5	6	12	23	10	5	7	22	45
*Mansonia septempunctata* ^[a]^	5	4	27	36	0	1	3	4	40
*Mansonia uniformis* ^[a,b,e,f]^	0	2	1	3	0	0	0	0	3
*Tripteroides atripes* ^[?]^	0	1	0	1	0	0	0	0	1
*Tripteroides magnesianus* ^[?]^	4	2	0	6	6	0	0	6	12
*Tripteroides* sp.	4	6	0	10	3	0	0	3	13
*Verrallina carmenti* ^[a]^	20	4	0	24	13	27	3	43	67
*Verrallina funerea* ^[a,b,g]^	0	1	0	1	0	0	0	0	1
*Verrallina lineata* ^[a]^	353	193	71	617	31	41	10	82	699
Total	1200	1152	1139	3491	384	339	290	1013	4504

^a^ alphaviruses (may cause Barmah Forest, chikungunya, Ross River, Sindbis)

^b^ flaviviruses (may cause dengue, Murray Valley-, Australian-, Kunjin- & Japanese encephalitis; yellow fever, Edge Hill, West Nile)

^c^ *Plasmodium* spp. (may cause human and/or avian malaria)

^d^ *Dirofilaria immitus* (may cause dog heartworms)

^e^ *Wuchereria bancrofti* (may cause filariasis in humans)

^f^ *Brugia malayi* (may cause filariasis in humans)

^g^ *Orbivirus* spp. (may cause epizootic hemorrhagic disease in ruminants & macropods)

^?^ unknown

### Mosquito community composition

**Fig. 5:**
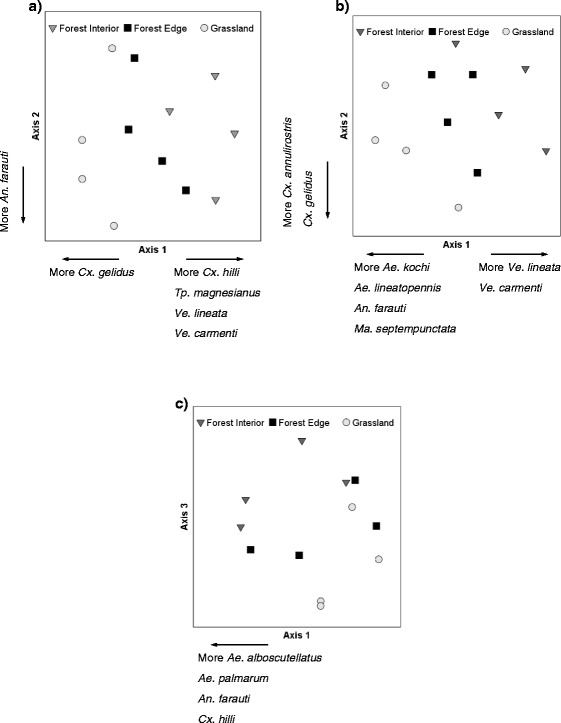
Ordination analyses of mosquito communities for habitats and seasons. Ordination analyses (NMS) show that the mosquito community varied strongly in response to habitat type. Forest interior sites are distinctly different from grassland sites when (**a**) the data for the wet season and dry season were combined, the data for the wet season (**b**) and the dry season (**c**) (only two of the three dimensions obtained in the analysis are displayed) were analysed separately

Using ordination analyses, we examined the mosquito community composition in three ways. First, we combined data for the wet and dry season (Fig. 5a) and determined that the mosquito community varied in response to habitat type, especially between forest interior and grassland sites (PerMANOVA: *pseudo F* = 2.164, *P* = 0.028). The NMS Axis 1 (which captured 45 % of the total variation) and Axis 2 (capturing 42 % of the variation) both discriminated mosquito communities in forest interiors that differed from those in grasslands. Of the 22 species examined, 6 were significantly correlated with these axes (Table 3). Secondly, when we explored the influence of seasonality on the community structure and found an identical and significant pattern of rainforest and grassland separation: (wet season PerMANOVA: *pseudo F* = 2.274, *P* = 0.028) and (dry season PerMANOVA: *pseudo F* = 1.608, *P* = 0.026). The wet season ordination (Fig. 5b) explained 91 % of the variation in the data (Axis 1: 55 %, Axis 2: 36 %), with 21 species examined and 8 significantly correlated with these axes (Table 3). The dry season analysis (Fig. 5c) explained 93 % of the total variation in the data set with 6 out of 19 species significantly correlated with these axes (Table 3). In summary, all three ordination analyses revealed that the mosquito communities were different between forest interior and grassland sites and that an overlap in species composition existed between forest interior and forest edge sites and between grassland and forest edge sites.

**Table 3:** Pearson correlations for mosquito species with two or three ordination axes produced by nonmetric multidimensional scaling (NMS). Correlation values in boldface were significant (*P* < 0.005) using a Bonferroni-corrected alpha value. *Mosquito community (all species were used for the analysis); ** Vector Community (only species capable of vectoring alpha-, flaviviruses and protozoans were considered for the analysis); *** All Seasons (wet and dry seasons combined)

	Mosquito Community*							Vector Community**	
	All Seasons***		Wet Season		Dry Season			All Seasons***	
Species	Axis 1	Axis 2	Axis 1	Axis 2	Axis 1	Axis 2	Axis 3	Axis 1	Axis 2
*Aedes alboscutellatus*					**-0.871**	0.319	0.246		
*Aedes kochi*			**-0.784**	0.096				**-0.811**	-0.062
*Aedes lineatopennis*			**-0.762**	0.281				**-0.767**	0.248
*Aedes palmarum*					**-0.866**	-0.088	-0.103		
*Aedes quinquelineatus*					**-0.796**	0.387	0.087		
*Aedes vigilax*					0.15	**0.787**	0.023		
*Anopheles farauti*	-0.37	**-0.854**	**-0.769**	0.47				**-0.879**	0.325
*Cq. nr. crassipes*								0.081	**0.786**
*Culex annulirostris*			0.039	**-0.793**					
*Culex gelidus*	**-0.845**	0.441	-0.393	**-0.791**				-0.289	**-0.902**
*Culex hilli*	**0.799**	0.235			**-0.839**	0.005	0.243		
*Mansonia septempunctata*			**-0.837**	0.131				**-0.865**	0.013
*Tripteroides magnesianus*	**0.792**	-0.278							
*Verrallina carmenti*	**0.775**	-0.508	**0.77**	0.257				0.183	**0.921**
*Verrallina lineata*	**0.815**	0.357	**0.777**	0.14	-0.194	**-0.789**	0.529	**0.82**	0.301

### Mosquito vector community

**Fig. 6:**
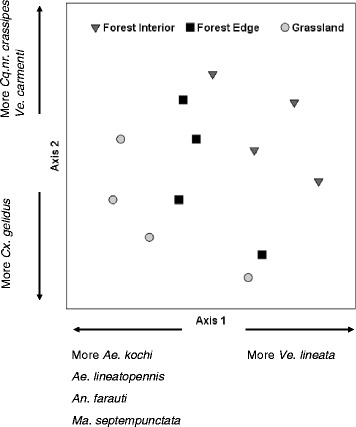
Ordination analysis of the vector community. Ordination analysis (NMS) of the vector community displays a distinctly different species composition for forest interior and grassland sites

**Fig. 7:**
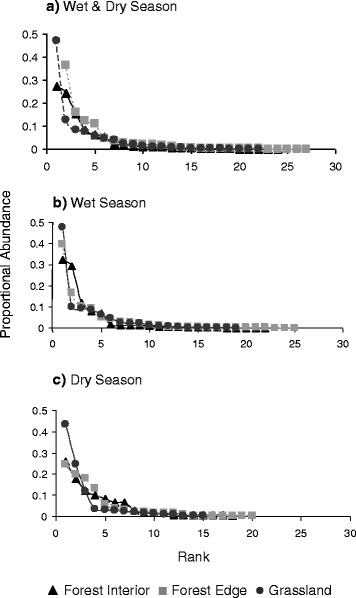
Rank-abundance diagrams for the diversity of mosquito species. Rank-abundance diagrams displaying the diversity of mosquito species in the three habitats; taking into account not just the number of species (richness) but also the distribution of individuals among species (evenness). Overall (**a**) (wet and dry season combined) forest interior had 2 dominant species; forest edge and grassland had one dominant species. In the wet season (**b**) two dominant species were discovered in the forest interior but only one dominant species in both grassland and forest edge. During the dry season sampling (**c**) one dominant species was captured in the grassland and forest edge and two species dominated the forest interior

We further examined the habitat preference of known disease vectors– mosquito species capable of transmitting alpha-, flaviviruses and protozoans and found a significant difference between rainforest interior and grassland sites (Fig. 6). The two ordination axes collectively explained 87.1 % of the total variation with 8 species significantly correlated with these axes (PerMANOVA: *pseudo F* = 2.502, *P* = 0.029) (Table 3). Notably, we observed more known disease vectors in grasslands than in forest habitats. We further investigated the mosquito community structure of each habitat type by examining species dominance using rank abundance diagrams (Fig. 7a, b and c). Overall, *Cx. annulirostris* was the most dominant species. During the wet season, it dominated grasslands and forest edges, and shared dominance of rainforest interiors with *Ve. lineata*. In the dry season, *Cx. annulirostris* continued to dominate the grasslands but shared dominance of forest edges with the rainforest species *Ae. notoscriptus*. Only one species dominated rainforest interior (*Ae. notoscriptus*).

### Mosquito characteristics

**Fig. 8:**
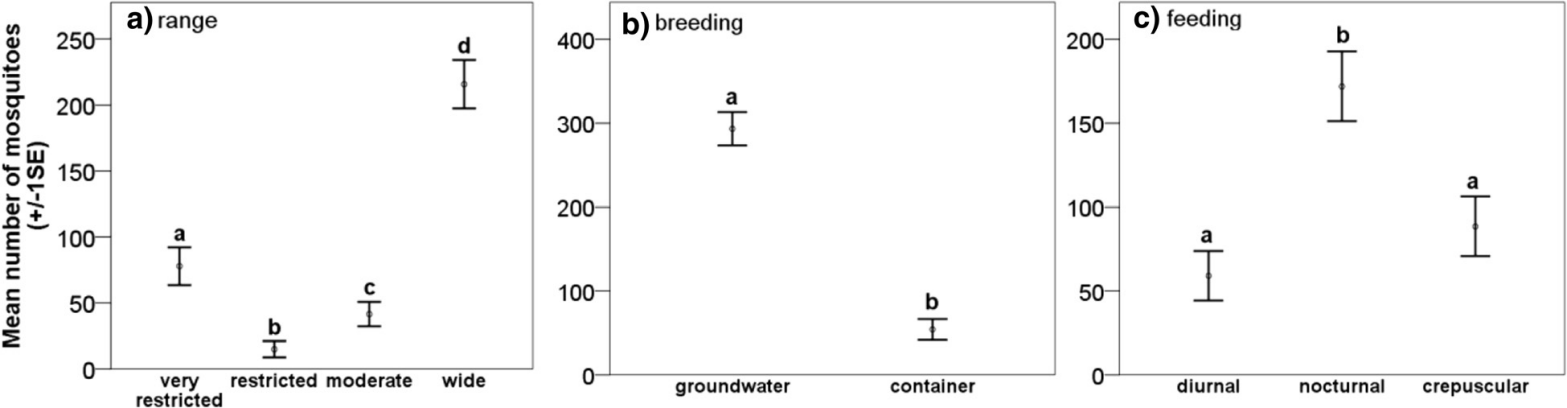
Mosquito characteristics. Mean number of captured mosquito groups in regards to geographical range, breeding habitat and time of feeding in northern Australia. Most captures were from the mosquitoes which have a wide distribution (**a**), use groundwater environments for depositing eggs (**b**) and blood-feed mainly during the night (**c**). Different letters denote significant differences

We found that ecological and biological characteristics strongly influenced mosquito captures in the study area. Commonly captured mosquitoes (>40 individuals) had a wide geographical range (Fig. 8a), were ground water breeders (Fig. 8b) and nocturnal blood-feeders (Fig. 8c). The mosquito group which has a wide geographical range in Australia was captured nearly three times more than the second most common group (the very restricted range group) (2-way ANOVA *F*_(3,36)_ = 75.045, *P* < 0.0001). All groups were significantly different from each other (Tukey HSD *post-hoc* tests *P* < 0.018). No differences in mean captures occurred in the three habitats (2-way ANOVA *F*_(2,36)_ = 0.163, *P* = 0.85); however, there was an interaction effect between groups and habitat (2-way ANOVA *F*_(6,36)_ = 5.286, *P* = 0.001), with rainforest habitats supporting more species with a very restricted range than the other habitats. Mosquitoes which belong to the group of groundwater breeders were captured six times more often than container breeders (2-way ANOVA *F*_(1,18)_ = 106.086, *P* < 0.0001). Habitat type had no influence on mean captures (2-way ANOVA *F*_(2,18)_ = 0.173, *P* = 0.842) and there was no interaction effect between groups and habitat type (2-way ANOVA *F*_(2,18)_ = 2.070, *P* = 0.155). We found that there was a significant difference of time of feeding amongst the collected mosquitoes (2-way ANOVA *F*_(2,27)_ = 12.459, *P* < 0.0001). Mosquitoes captured in this study blood-feed predominantly at night (54 %), followed by the crepuscular group (28 %) and the diurnal group (18 %). Nocturnal and diurnal feeders differed significantly (Tukey HSD *post-hoc* test *P* < 0.0001) as did nocturnal and crepuscular feeders (Tukey HSD *post-hoc* test *P* = 0.004). There was no significant difference between the diurnal and crepuscular group (Tukey HSD *post-hoc* test *P* = 0.431). Habitat had no influence on the mean captures (2-way ANOVA *F*_(2,27)_ = 2.522, *P* = 0.099) and no interaction effect was detected (2-way ANOVA *F*_(4,27)_ = 1.613, *P* = 0.200).

## Discussion

Communities in naturally-occurring ecotones are often an integration of species from adjacent habitats [44]. We observed a similar pattern in mosquito communities although across an anthropogenic disturbance gradient from tropical grassland to rainforest, where forest edges supported mosquito species from both habitats. This landscape pattern continued throughout the year, despite a seasonal influence on mosquito abundances. The majority of the mosquitoes at our study area showed significant traits such as groundwater breeders, nocturnal blood-feeders and a wide geographic distribution across Australia. Our analysis of disease risk found a significant difference between grassland and forest habitat, with a greater abundance of known disease vectors in grasslands.

Forest edges acting as ecotones, may produce a novel juxtaposition of mosquito communities which could have wide-reaching consequences for mosquito-borne disease transmission [45] as an increase of endemic viruses is more likely to occur in disturbed habitats than in pristine primary forests. For instance, Junglen et al. [46] found that the mosquito genera *Aedes*, *Anopheles* and *Culex* were more commonly encountered in disturbed habitats and contained more virus isolates than forest mosquitoes.

We found edges are hot beds of potentially disease vectoring species and that they have an important role in facilitating disease transmission across the landscape. Disturbed and degraded habitats are avoided by numerous forest species [47–49]. However some, especially invasive and generalist species seem to prefer these habitats [50, 51] which may explain why the mosquito community composition was significantly different between man-made grasslands and forest interior sites in our study. A previous, short-term study we conducted in the same area [41] also showed that grasslands supported a markedly different community to inside forests. These distinct differences in mosquito community composition between grassland and forest interior sites may be because certain mosquito species (e.g. *An. farauti* and *Cx. gelidus*) find open habitats such as grasslands more attractive than closed habitats.

The question that then arises is “why do some mosquitoes prefer open habitats to closed habitats”? The answer might be that open and disturbed habitats feature environmental characteristics such as higher temperatures and light levels, higher pH- and lower salt levels, which can accelerate larval growth and increase larval survivorship. These changing environmental conditions also contribute to faster growth of algae – an important food source for mosquito larvae [4, 8–11].

It was not unexpected that more mosquitoes and species were captured in the wet season compared to the dry season. Mosquitoes are commonly associated with rainfall [3, 52]. However, it was surprising to find that some important disease vectors such as *Ae. notoscriptus*, *Ae. vigilax, An. farauti* and *Cx. annulirostris* in our study area were able to persist in the dry season. This suggests that disease transmission could potentially occur at any time of the year.

Previous studies have found that refugia with favourable microclimatic conditions may help a mosquito population to persist year round. For example, Hightower et al. [53] found that malaria vectors (*Anopheles* spp.) in Kenya retreat to vegetation around permanent water bodies during the dry season, allowing for year round reproduction. Water-filled tree holes and abandoned snail shells can also be used for oviposition [54]. Additionally, anthropogenic changes to the environment like the construction of irrigation areas and dams allow mosquitoes to breed regardless of seasons [55–58]. Jardine et al. [59] demonstrated that the Ord River Irrigation Area in Western Australia is responsible for mosquitoes breeding even during the driest month of the year. For example, *Cx. annulirostris*, the most abundant species in our study and responsible for transmitting numerous viruses, (Ross River, Kunjin, Murray Valley and Japanese encephalitis) [60, 61] was found to be very active during the dry season [59].

There may be other mechanisms that contribute to the year-round persistence of mosquitoes. Biological, physiological and ecological attributes such as desiccation-resistant eggs, egg dormancy, diapause and larval development in moist soil, leaf litter or plant axils and adult and larvae hibernation, aestivation, quiescence and diapause could be mechanisms for survival in environments with seasonal periods of droughts [54, 62–65]. Additionally, some adult mosquitoes are able to aestivate by gonotrophic dissociation (the pausing of egg production despite acquiring several blood meals) or by performing diapause [66–70]. Lastly, man-made and natural shelters may provide refugia for inactive parous (having laid eggs at least once) female mosquitoes [71]. Captured mosquitoes from our study most likely used one or a combination of the strategies outlined above to persist year-round. For example, *Ae. notoscriptus* has both desiccation-resistant eggs and the ability to perform hibernation and diapause during larval and adult life stages [72, 73]. However, the eggs of *Cx. annulirostris*, the most dominant species in this study, are prone to desiccation [25]; but adults can perform diapause [68] and are known to lay eggs in shallow, grassy pools only hours after a rainfall event [74]. This is in accordance with the findings of this study as most *Cx. annulirostris* were captured in grasslands.

We expected that mosquito species richness would be highest in forest interior habitats as tropical forests often support greater diversity within forest interiors and decline along forest edges [47–49]. However, this pattern was not observed in this study for mosquito diversity. In contrast, the number of species was quite evenly distributed across the three habitat types. Even though the sampling effort was sufficient, rare species, most notably canopy specialists, could be absent from our samples as a consequence of the trap height used (~1.5 m). More extensive sampling at different vertical strata would most likely increase the probability of capturing such rare species in future studies [75].

We believe that it is crucial to comprehend the impacts of landscape disturbance, especially the impacts of deforestation and forest fragmentation, on mosquito communities. Commonly-captured mosquito species in our study were found to be widespread in geographic distribution, ground breeders and nocturnal feeders. We consider the current distribution of these species to be the result of land clearing and agriculture (predominantly sugar cane). Prior to European arrival, tropical rainforests, open eucalypt forests and estuarine vegetation blanketed the region [76], and the mosquito communities probably included species that were more restricted in distribution to the different habitats.

Our observations of changing mosquito communities in response to land use support the concept that new or re-emergent mosquito-borne diseases could arise in areas where land use changes occur. Disease transmission will almost certainly emerge via common mosquito species captured across all habitats and seasons (e.g. *Cx. annulirostris*) or through species more prolific within open habitats, such as man-made grasslands (e.g. *An. farauti, Cx. gelidus, Ma. septempunctata*) [41]. Previous studies from tropical areas have already demonstrated that human-induced land use changes, such as deforestation, are responsible for a rise in important disease vectors such as Anophelinae and Aedinae [3, 77–79]. However, our study is the first to suggest that common species and species which are potential disease vectors can maintain populations across a land use gradient throughout the year.

## Conclusions

We demonstrated that the mosquito community in north Queensland strongly responded to anthropogenic land use changes. Our results displayed that there is a diverse mosquito community in tropical Australia, but more importantly that the community composition varies considerably between forests and disturbed habitats. Additionally, most disease transmitting species predominantly occur in grasslands created by humans. This strong influence of anthropogenic land use change on mosquito communities could have potential implications for pathogen transmission to humans and wildlife. We also found that vectors of mosquito-borne diseases, such as *Cx. annulirostris*, can persist all year round, further increasing disease risk. Considering that human-induced land use changes and human population growth are advancing rapidly in tropical regions, it is of the utmost importance to predict future disease risk. Historically, mosquito studies have been predominantly focused on single species life-cycles in association with the urban environment, we suggest further ecological studies are necessary to understand how land use changes will influence disease dynamics of the whole community in order to predict and prevent future health threats.

## Abbreviations

ANOVA, analysis of variance; NMS, nonmetric multidimensional scaling ordination; PerManova, permutational multivariate analysis of variance; Tukey HSD, Tukey Honest Significant Difference *post-hoc* test.

## Additional file

Additional file 1:
**Table S1.** Abundance. **Table S2.** Dry Season 1. **Table S3.** Dry Season 2. **Table S4.** Wet Season 1. **Table S5.** Wet Season 2 (XLS 83.5 kb)
